# Factors associated with the exposure of vaccines to adverse temperature conditions: the case of North West region, Cameroon

**DOI:** 10.1186/s13104-015-1257-y

**Published:** 2015-06-30

**Authors:** Martin Ndinakie Yakum, Jérôme Ateudjieu, Fida Ramsina Pélagie, Ebile Akoh Walter, Pierre Watcho

**Affiliations:** Department of Biomedical Sciences, University of Dschang, P.O. Box 067, Dschang, Cameroon; Division of Health Operations Research, Ministry of Public Health, Yaoundé, Cameroon; Meilleur accès aux soins de santé (M.A.SANTE), P.O. Box 33490, Yaoundé, Cameroon; Bamenda health district service, Bamenda, Cameroon

**Keywords:** Vaccine, Cold chain, EPI, Immunization, Abnormal-temperature, Determinants, North-West, Cameroon

## Abstract

**Background:**

Adverse temperature recording in vaccine’s cold chain is a major issue worldwide and this condition is known to compromise the quality of vaccines very rapidly. In Cameroon, with tropical climate, vaccines exposure to abnormal temperatures is very common. This study was conducted to identify factors associated to abnormal temperature recording in cold chain in the North West region.

**Results:**

A total of 65 vaccinating health facilities were visited for the study from eight health districts. Concerning type of health facility, 48 (73.8%) of the health facilities were governmental facilities. About 50 (76.0%) of the facilities had a functional thermometer. Among the 50 health facilities with functional thermometer, abnormal temperatures were registered in 10 (20%) health facilities during data collection and 12 (24%) in the 2 months preceding collection. Factor significantly associated with abnormal temperature recording was the absence of an alternative power source (OR = 6.5, p = 0.03).

**Conclusion:**

The absence of an alternative source of power was significantly associated with abnormal temperature exposure in the 2 months preceding data collection. To improve on the quality of vaccines administered in North West region, each vaccinating health facility must have at least two sources of power supply.

## Background

Immunization is the most cost-effective preventive health intervention presently known in modern medicine [1, 2]. To achieve full benefits of immunization, it is necessary to ensure high coverage, availability of potent vaccines, and timely delivery of scheduled immunizations [3, 4]. Vaccines are biological substances that gradually lose their potencies with time [5]. This loss of potency can be accelerated when exposed to excessive heat, freeze, or light [5, 6]. The conservation of vaccine’s potency from the manufacturer down to users is still very challenging. The exposure of vaccines to adverse temperature conditions does not only nullify the immunization effect, but also induces adverse events following immunization [7–10]. Therefore, vaccines must be handled with care and a lot of attentions to ensure their quality and consequently full immunization benefit.

The role of cold chain is at the center of the Expanded Program on Immunization (EPI)’s activities since it maintains vaccines at a narrowed temperature environment (necessary for potency conservation) from the manufacturer down to the users [1, 2]. However, within the cold chain, maintaining the temperature is never really easy due to varying environmental (weather, climate, and human activities) conditions [11]. Also, the equipment of the cold chain and power supply might register failure at any given time [2]. Consequently, continuous monitoring of the cold chain is indispensable so that vaccines exposure to adverse temperatures must be noticed early enough to take action. Cold chain failure and frequent exposure of vaccines to adverse temperature conditions have been documented in several parts of the world [11–15].

In developed countries, exposure to freezing temperatures is most frequent [15] conversely to developing countries where exposure to overheating is the most frequent [12, 13]. It is believed that; tropical climates, unreliable power sources, and limited resources (material, financial, and human) are factors responsible for the frequent exposure of vaccine to overheating temperature conditions in developing countries [1, 2, 5].

In Cameroon, outbreaks of vaccine preventable diseases are common and vaccine exposure to abnormal temperatures has been documented. The North West region is one of the regions constantly in epidemic of vaccine preventable diseases [16]. Also, high rate of exposure of vaccines to abnormal temperatures has equally been documented here [12]. This paper attempts to evaluate factors associated with vaccine exposure to adverse temperature condition at primary health care and district levels in the North West region of Cameroon.

## Methods

### Study design

It was a cross sectional study targeting primary health care centers and district cold chains and was conducted in December 2013. Sampling was done at multiple levels and data collected with a preconceived and validated grid by observation and consultation of the vaccine-related documents. Associated factors were tested by calculating the odds ratio (OR), confidence interval and *P* value with simple logistic regression and potential confounders controlled on a multiple logistic regression.

### Setting

The North West Region of Cameroon had an estimated total population of 1,901,579 inhabitants for 2013 unevenly distributed among 19 health districts and 218 health areas. In total, there are 12 rural and 7 urban health districts. It lies between latitudes 5°40′ and 7° to the North of the equator, and between longitudes 9°45 and 11°10′ to the East of the Meridian. It is bordered to the south west by the South West region, to the south by West region, to the east by Adamawa region, and to the north by the Federal Republic of Nigeria. Concerning the weather temperatures during the 2 months targeted by this study (November and December 2013), the average temperature for the month of November was 19.4°C (min = 14.9°C, max = 23.8°C) and that’s of December was 19.5°C (min = 14.4°C, max = 24.6°C). Concerning power supply, about 80% of the region has access to electricity.

### Ethical statement and authorization

The study targeted immunization’s cold chain of health facilities and did not have anything to do with human subjects or their biological or personal information. Therefore, ethical approval was not requested. The authorization to carry out the research was obtained from the regional delegate of Public Health for the North West region.

### Study population

**Figure 1:**
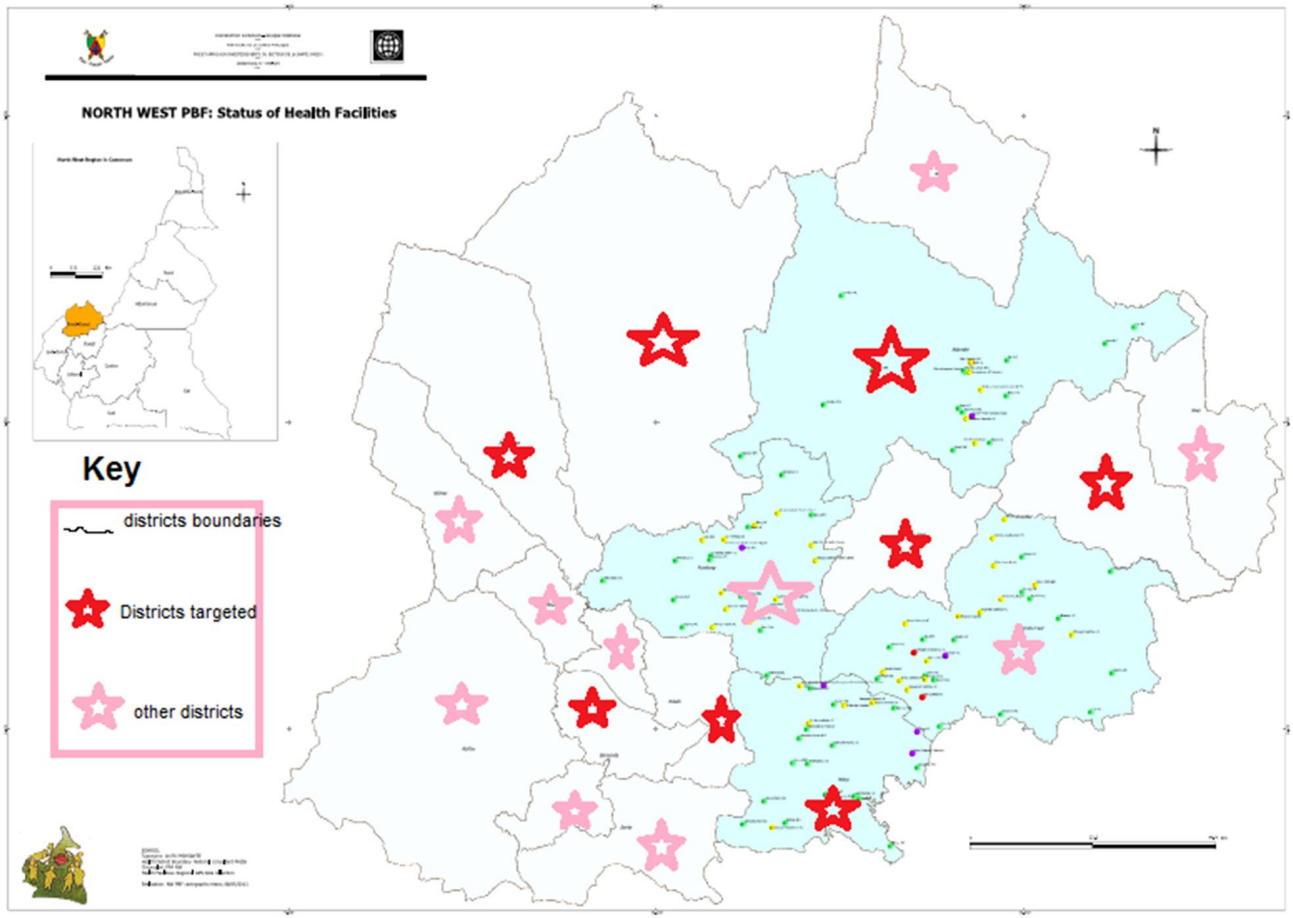
Map of Cameroon showing the North West region and the health districts. The map and the permission to use it were obtained from the regional delegation of public health for the North West region and the delegation is informed that the figure will be published in an open access journal.

The study involved vaccinating health facility in the selected health districts. Eight health districts were selected for the study. These included: Bamenda, Benakuma, Kumbo West, Ndop, Ndu, Nkambe, Tubah, and Wum Health Districts. Figure 1 shows the selected health district on the Map of the North West region, Cameroon

The Cameroon health system is divided into 3 levels: the central level, represented by the ministry of health; the intermediate level, represented by the regional delegations of public health; and the Peripheral level, represented by the health districts. The health district is managed by the district health service (DHS) which is headed by a district medical officer. A health district is made up of many health areas and each health area has at least one primary healthcare center which is responsible for delivering health care services to the population of the health area. The primary health care centers are categorized into: integrated health centers (IHC) headed by a senior nurse, Sub-divisional hospital headed by a medical doctor or district hospital which is headed by a medical director. Concerning cold chain management, this is ensured at the primary health care center by the head, but this function can be assigned to one of the personnel. The national guideline recommends that a temperature chart should be pasted on all vaccine refrigerators. The personnel responsible of cold chain monitoring reads and records vaccine temperature twice daily (morning and evening) on the chart. Also, all power interruptions are indicated on the chart. It should be noted that this chart is designed to collect information for 1 year. Consequently, the chart is replaced at the beginning of each year and the previous filled one archived in the health facility archiving system.

### Sampling

All health districts of the North West were first of all grouped into 7 urban and 12 rural districts. Eight (42%) of these health districts, that is, three urban and five rural districts were selected from their respective groups by simple random sampling technique. In each selected district, the list of all health facilities involved in the immunization activities was made and seven selected by systematic sampling technique. Also, the district’s immunization cold chain system was evaluated. The sampling procedure was adopted to ensure the representative of the sample and to ensure the attainment of the sample size (estimated at 60) needed for the study.

### Data collection

Data were collected with a preconceived and validated grid. Data collected were on the availability and functioning of vaccine refrigerator, present of cold chain contingency plan, presence of a source of power and an alternative source of power supply, presence of temperature recording sheet, presence of a functional thermometer in the vaccine fridge, presence of the national guidelines on immunization, the temperature at the moment of data collection. A cold chain contingency plan is a plan or procedure that will take effect if emergencies such as power interruption and refrigerator breakdown occur. It is also called emergency plan and the national guidelines recommends it to all vaccinating centers. In health facilities without a functional thermometer, data on temperature exposure could not be obtained but all other data were collected.

### Data analysis

Data were analyzed with EpiInfo 3.5.4. In the first place, data forms were checked corrected and validated before entering into the computer. The entered data were further cleaned and analyzed. The availability and the functioning of cold chain materials were evaluated by calculating proportions such as the proportion of vaccine fridge having a functional thermometer, proportion of health facilities having a cold chain contingency plan. Also, the main outcome of interest was the abnormal temperature recorded during 2 months preceding data collection. Vaccines were said to have been exposed to adverse temperature conditions if one temperature recorded in the 2 months preceding data collection was greater than +8°C or less than +2°C; else, the vaccines were said not exposed to adverse temperature conditions. Two months period was just the time chosen by the research team to evaluate the cold chain. The association of adverse temperature exposure with some potential factors was estimated by calculating the OR, CI, and the P value using a simple logistic regression. Potential confounder, number of refrigerator break down during the 2 months was controlled in a multiple logistic regression to obtain the adjusted OR. The confidence intervals were calculated at 95% confidence.

## Results

### Characteristics of health facilities

A total of 65 vaccinating health facilities were visited for the study from 8 Health Districts. Concerning type of health facility, 48 (73.8 [61.5–84.0] %) of the health facilities were governmental facilities and 40 (61.5 [48.6–73.3] %) were Integrated Health Centers (IHC). All district cold chains in the eight District Health Services (DHS) were evaluated.

### Availability and functioning of cold chain

Table 1 below presents the availability and functioning of cold chain equipment and resources in the targeted health facilities.

**Table 1:** Availability and functioning of cold chain materials and equipment

Equipment and resources	Frequency (%)			
	Total	DHS (n = 8)	IHC (n = 40)	Others (n = 17)
Vaccine refrigerator (n = 65)				
Present	57 (87.7)	8 (100.0)	36 (90.0)	13 (76.5)
Functioning	53 (81.5)	8 (100.0)	32 (80.0)	13 (76.5)
Thermometer in functional refrigerators (n = 53)				
Present	51 (96.2)	8 (100.0)	30 (93.8)	13(100.0)
Functioning	50 (94.3)	7 (87.5)	30 (93.8)	13 (100.0)
Temperature sheet on functional refrigerators (n = 53)				
Present	50 (94.3)	6 (75.0)	31 (96.9)	13 (100.0)
Up-to-date	25 (47.2)	3 (37.5)	16 (50.0)	6 (46.2)
Cold chain contingency plan present (n = 57)	*12 (21.1)*	*0 (0.0)*	*10 (27.8)*	*2 (15.4)*
National immunization guidelines present (n = 65)	*41 (63.1)*	*5 (62.5)*	*28 (70.0)*	*8 (47.1)*
One personnel assigned to monitor cold chain (65)	*50 (76.9)*	*8 (100.0)*	*28 (70.0)*	*14 (82.4)*
Power sources (n = 65)				
Electricity	53 (81.5)	8 (100.0)	30 (75.0)	15 (88.2)
Presence of at least two sources	41 (63.1)	4 (50.0)	24 (60.0)	13 (76.5)
Cold boxes/vaccine carriers present	65 (100.0)	8 (100.0)	40 (100.0)	17 (100.0)
Presence of icepack	65 (100.0)	8 (100.0)	40 (100.0)	17 (100.0)

Italic values indicate statistical significance (*p* ≤ 0.05).

### Temperature condition of vaccine

Among the 50 health facilities that had a functioning thermometer in a vaccine fridge, 10 (20 [10.0–33.3] %) of them had a thermometer reading out of the optimal vaccine storage temperature of +2°C to +8°C at the moment of data collection. Exposure to temperature higher than 8°C was more frequent 6 (12%) than the exposure to temperature less than 2°C 4 (8%). Furthermore, from the records of the 2 months preceding data collection, 12 (24.0 [13.1–38.2] %) health facilities had recorded at least one temperature out of the optimal vaccine storage temperature of +2°C to +8°C. Similarly, exposure to overheat was more frequent. A total of 12 (24%) vaccine fridges were exposed to overheating (temperature higher than 8°C) and 6 (12%) exposed to cold (temperature lower than +2°C) in the two previous months to data collection. It is worth noting that all the vaccine refrigerators that were exposed to cold during the two months were equally exposed to overheating.

### Factors associated with the exposure of vaccines to adverse temperature conditions

Table 2 presents the potential factor associated with the exposure of vaccine to abnormal temperature.

**Table 2:** Potential factor associated with the exposure of vaccine to abnormal temperature

Factor	Simple logistic regression			Multiple logistic regression		
	OR	CI	P value	OR_adj_	CI	P value_adj_
Absence of a cold chain contingency plan	1.8	0.33–9.59	0.4990	1.5	0.28–8.53	0.6220
Absence of guidelines	1.0	0.24–4.11	0.6230	1.4	0.30–6.34	0.6792
Absence of an alternative power source	3.5	0.83–14.69	0.0843	6.5	1.14–37.05	0.0352*
Was not supervised within 2 months	1.6	0.37–6.53	0.4017	2.8	0.59–13.36	0.1874

* Statistically significant association.

It should be noted that, none of the 10 health facilities that recorded abnormal temperature during the 2 months preceding data collection had a cold chain contingency plan whereas 12 (30%) of those with correct temperature had a cold chain contingency plan.

## Discussion

This paper attempts to identify factors associated with abnormal temperature recording in vaccine’s cold chain. It might save as a reference, helping to identify simple practices capable of improving the quality of vaccine.

It came out from the results that 24% of the vaccine refrigerators were exposed sub-optimal temperatures within a 2 months period. The problem of overheating was documented in all refrigerators that were exposed to sub-optimal temperatures whereas only 67% of them were exposed to freezing temperatures. The absence of an alternative source of power supply (OR = 6.5, P = 0.03 was associated with vaccine exposure to sub-optimal temperatures.

The exposure of vaccines to sub-optimal temperatures indicated in this paper is a worldwide problem and has been documented in many parts of the world [11–16]. This problem might be caused by factors related to the cold chain equipment, personnel, and/or power supplies [17, 18]. The rate of exposure to sub-optimal temperatures may even be higher than the 24% documented here since exposure during transportation was not evaluated. For example, about 18.5% of health facility did not have a functional vaccine refrigerator and this implies they have to transport vaccines from and to the closest health facility with a functional cold chain before and after each immunization session respectively [2, 11]. This increases the chance of exposing vaccines to heat or freezing, and consequently reduces vaccine’s potency and increases the wastage rate [3, 11, 12]. Many research papers hold the opinion that freezing of vaccine is very common and more likely during transportation than overheating [3]. The paper proposes that the lack of an alternative source of power supply is associated with vaccines exposure to adverse temperature conditions. It was documented in a study in Nigeria that irregular power supply of health facilities and absence of standby generator were major risk factors of loss of vaccine potency [19]. Though not statistically significant here, the use of vaccine contingency plan is recommended by national and international guidelines on immunization [1–5] and supervision has showed to increase the adverse event following immunization detection and reporting rates in Cameroon [10]. Therefore, to minimize the rate of exposure of vaccine to adverse temperature conditions, all vaccinating centers should be provided with a vaccine refrigerator and an alternative power supply [20]. Also, these health facilities should be supervised regularly stressing on the use of contingency plan in all centers [21].

The need for innovative technology in cold chain monitoring that can help reduce the rate of vaccine exposure to adverse temperatures or that can lead to the production of thermo-stable vaccines has been well established [22–29]. Innovating technologies that can help target the weakness of cold chain identified in this paper include: production of thermo-stable vaccines [11, 30], computerization of cold chain monitoring, the use of electronic temperature monitor with alarm, and the production of long-life cold chain equipment [31]. A systematic review has documented the possibility to produce thermo-stable vaccines at low cost from plants [30]. The computerization and electronic cold chain monitoring and the production of long-life cold chain have equally been demonstrated [31]. However, the production of long-life cold chain can only prevent overheating but not freezing. The production of thermo-stable vaccines has been demonstrated only in some cases like the Influenza virus vaccine [30] and there is not yet enough evidence that it can also be done for other vaccines. Also, the computerization of cold chain monitoring and the use of electronic monitor with alarm have been tested and used in developed countries like the USA [31]. Therefore, all these innovating technologies to improve on cold chain still need to be tested in resource-limited settings to enable their universal benefits.

The shortcomings of this paper include the fact that data were collected on vaccine only at storage and not during transportation; temperature records used were those registered twice daily which cannot actually tell how many times adverse temperatures occurred; and the fact that some health facilities refused participating in the study. However, Health districts and health facilities were selected randomly and the surveyors were well trained before the start of data collection. The results of this study can therefore be inferred to the whole of the North West region.

## Conclusion

Exposure of vaccines to adverse temperature conditions is a major problem in the North West region of Cameroon. This problem is not only grounded at health facilities but also at district health services which is supposed to oversee and supervise the immunization related activities of the health facilities. The lack of an alternative source of power supply was significantly associated with adverse temperature exposure of vaccines. In order to reduce the rate of vaccine exposure to adverse temperatures, we recommend the following practices to the regional delegation of Public Health for the North West region and the various district health services:

- Ensure that each vaccinating health facility and district health services is provided with at least one stand-by source of power different from the main source.
- Supervise the health facility regularly, not more than 2 month per facility and insisting on the use of contingency plan in all the facilities.
- Appoint one health personnel to monitor the cold chain in each health facilities and district health services.

## Abbreviations

DHS: District Health Service
EPI: expanded programme on immunization
HF: health facility
IHC: Integrated Health Center
OR: odds ratio
USA: United States of America
